# Screening for *Lactobacillus plantarum* Strains That Possess Organophosphorus Pesticide-Degrading Activity and Metabolomic Analysis of Phorate Degradation

**DOI:** 10.3389/fmicb.2018.02048

**Published:** 2018-09-03

**Authors:** Changkun Li, Yuzhu Ma, Zhihui Mi, Rui Huo, Tingting Zhou, Huricha Hai, Lai-yu Kwok, Zhihong Sun, Yongfu Chen, Heping Zhang

**Affiliations:** ^1^Key Laboratory of Dairy Biotechnology and Engineering, Ministry of Education, Inner Mongolia Agricultural University, Huhhot, China; ^2^Key Laboratory of Dairy Products Processing, Ministry of Agriculture, Inner Mongolia Agricultural University, Huhhot, China

**Keywords:** *Lactobacillus plantarum*, organophosphorus pesticides, dimethoate, phorate, omethoate

## Abstract

This work performed a large scale assessment for organophosphorus pesticides (OPPs) degradation activity of 121 *Lactobacillus* (*L*.) *plantarum* strains. Six *L. plantarum* strains (P9, IMAU80110, IMAU40100, IMAU10585, IMAU10209, and IMAU80070) were found to possess high capacity of degrading three commonly used OPPs, namely dimethoate, phorate, and omethoate; and they were selected for more detailed characterization. Moreover, the three OPPs were mainly detected in the culture supernatants but not in the cell extracts, further confirming that the OPPs were degraded rather than absorbed by the cells. Among the six selected strains, P9 was most tolerant to gastrointestinal juices and bile. We thus used ultra-high performance liquid chromatography electron spray ionization coupled with time-of-flight mass spectrometry (UPLC/ESI-Q-TOF/MS) to generate the metabolomic profiles of the strain P9 growing in MRS medium with and without containing phorate. By using orthogonal partial least squares discriminant analysis, we identified some potential phorate-derived degradative products. This work has identified novel lactic acid bacteria resources for application in pesticide degradation. Our results also shed light on the phorate degradation mechanism by *L. plantarum* P9.

## Introduction

In China, organophosphorus pesticides (OPPs) are the most widely used chemical pesticides. The annual consumption of OPPs is around 3.00 × 10^5^ tons, accounting for about 72% of total pesticide application (Chu et al., 2018). However, only a minute proportion (roughly 0.1%) of the applied pesticides reaches the target pests; and the rest spreads through water, soil, and food to the ecosystem (Abdel-Halim et al., 2006; Karami-Mohajeri et al., 2017). Acute poisoning with OPPs is considered a global threat that causes more than 100,000 deaths a year (Karami-Mohajeri et al., 2017). OPPs may cause neurotransmitter disorders via inhibiting the acetylcholinesterase enzyme activity (Rezg et al., 2010). Further, OPPs accumulation significantly increases the level of liver free radicals and burdens in detoxification (Dhouib et al., 2015). The bioaccumulation of OPPs may also lead to other health issues, like allergic diseases (Yanagisawa et al., 2008; Fukuyama et al., 2011), ataxia and paralysis (Díaz-Resendiz et al., 2015), metabolic disruption (Debost-Legrand et al., 2016), type 2 diabetes (Rezg et al., 2010), and heart disease (Hung et al., 2015). Thus, the environmental pollution of OPPs poses serious health risk to humans and wildlife (Rezg et al., 2010).

The National Pesticide Information Center of United States listed chlorpyrifos, phorate, dimethoate, malathion, acephate, naled, dicrotophos, phosmet, diazinon, and azinphos-methyl as the most used OPPs (NPIC^1^). Residues of some OPPs are repeatedly reported in the terrestrial and aquatic food chains (Regueiro et al., 2015), e.g., dimethoate residues in soil (Liu et al., 2016), olives (Paíga et al., 2016), and apples (Szpyrka et al., 2015); phorate residues in green tea (Steiniger et al., 2010), livestock products (Rahman et al., 2016), and soil (Stoleru et al., 2015; Ramasubramanian and Paramasivam, 2016); and omethoate residues in various vegetables (Stoleru et al., 2015). Therefore, our work focused on identifying lactobacilli that could degrade these three OPPs, i.e., dimethoate, phorate, and omethoate. Bioremediation is a technology that applies microorganisms to detoxify and degrade pollutants. This technology has received increased attention as an effective and a reliable biotechnological approach to clean up pesticide polluted environments. The genera *Pseudomonas* (Gu et al., 2007) and *Paracoccus* (Xu et al., 2009) are the major microbes that have been successfully used in degrading OPPs in polluted environments. Owing to the increased food safety awareness of the public, natural decontamination methods like degrading toxic and hazardous substances in raw food materials or during food processing have received wide attention (Regueiro et al., 2015). However, only very few reports have investigated microbial OPPs degradation in food matrices. Lactic acid bacteria are “generally recognized as safe” microorganisms. Probiotic *Lactobacillus* (*L*.) may be potential microbes for reducing unavoidable pesticide absorption in humans and wildlife (Trinder et al., 2015). Some *Lactobacillus* strains possess natural ability to degrade pesticides *in vitro* and alleviate pesticide poisoning *in vivo*, e.g., in the fermentation of kimchi (Islam et al., 2010), fermented milk (Bo et al., 2011; Zhao and Wang, 2012; Zhou and Zhao, 2015), wheat slurry (Đorđević and Đorđević-Pejcìv, 2016), and corn silage (Zhang et al., 2016). Trinder et al. (2016) demonstrated the alleviation of OPPs poisoning by *L. plantarum* in Drosophila. Pesticide degradation is both strain- and pesticide-specific. Indeed, the growth of some microorganisms can be inhibited by specific pesticide (Lénárt et al., 2013; Harishankar et al., 2013). Therefore, in order to develop strategies in probiotics-based pesticide degradation in food matrices, it is important to screen for food-originated strains that are relatively resistant to the target pesticides.

Metabolomics is a sensitive technology which provides comprehensive and quantitative profiles of metabolites in a biological system; and liquid chromatography coupled with mass spectrometry (LC-MS) is one of the most widely-used analytical tools for untargeted metabolomic studies (Zhang N. et al., 2012). Such approach has been successfully applied to identify the metabolites released during fenhexamid degradation by *L. casei* (Lénárt et al., 2013) and characterize the plasma metabolomes of rats exposed to four OPPs (Du et al., 2014).

Since *L. plantarum* has been shown to alleviate toxicity of OPPs *in vivo*, this work aimed to screen OPPs-degrading strains from a bacterial culture collection of 121 *L. plantarum* strains. In order to ensure that the screened strains could be developed as potential probiotics, we also assayed the tolerance of selected bacteria to simulated gastric juice and bile. Finally, the mechanism of phorate degradation was further explored using a metabolomic approach.

## Materials and Methods

### Bacterial Isolates and Reagents

One hundred and twenty-one isolates of *L. plantarum* were obtained from the Lactic Acid Bacteria Culture Collection (LABCC) of the Key Laboratory of Dairy Biotechnology and Engineering, Inner Mongolia Agricultural University. All isolates were originated from traditional fermented foods and were identified as *L. plantarum* using a combination of traditional microbial identification methods in combination with 16S ribosomal RNA (rRNA) gene sequence analysis; their 16S rRNA gene sequences were submitted to GenBank (NCBI) (Zhang H.P. et al., 2012). All isolates were stored long-term in a skimmed milk medium (SMM, NZMP LTD., Zealand) at -80°C. They were activated by cultivation in de Man, Rogosa, and Sharpe (MRS, Oxoid Ltd., England) broth at 37°C for 24 h prior to use in experiments.

Three OPPs standards, dimethoate (99.50%), omethoate (96.80%), and phorate (95.60%), were purchased from Sigma-Aldrich (Saint Louis, MO, United States). They were stored at 4°C before use. Initial stock solutions (2000 mg/L) of each pesticide were prepared by dissolving the pesticides in acetonitrile solution with 0.2% acetic acid. Working stock solutions (ranging from 0.0625 to 0.5 mg/L) of the individual pesticides were prepared by diluting the initial stock solutions with acetonitrile. HPLC gradient grade acetonitrile, acetone, methanol, formic acid, and dichloromethane were purchased from Fisher Scientific (VWR, Radnor, PA, United States).

### Large-Scale Screening of 121 *L. plantarum* Isolates for OPPs Degrading Activity

Each of the reactivated *L. plantarum* isolate was washed and resuspended in phosphate buffer solution (PBS) in a concentration of 1 × 10^9^ CFU/mL. One milliliter suspension of each *L. plantarum* isolate was inoculated into 100 g of MRS containing dimethoate, omethoate, and phorate (each of 0.5 mg/kg). The OPPs solutions were sterile filtered through 0.22 μm pore size membranes before being added to the MRS medium. Three replicates were prepared in parallel for each *L. plantarum* isolate. Briefly, 30 mL of *L. plantarum*- and pesticides-containing MRS cultures were shaken vigorously for 30 s, aliquoted into three sterile glass bottles, and incubated at 37°C for 24 h. The incubated cultures were centrifuged at 12,000 ×*g* for 10 min. The bacterial pellets and supernatants were separately collected. The supernatants were filtered through a sterile 0.22 μm pore size membrane and stored at -20°C prior to testing for OPPs degradation using gas chromatography mass spectrometry (GC-MS). Aliquots of samples were also separately stored for determining the phosphatase activity. A blank control without *L. plantarum* inoculation was prepared in parallel for calculation of the percentage of OPPs recovery. The pesticide degradation and recovery were calculated according to the following formula:

$$\begin{array}{c} \text{Degradation rate }(\%)=(0.5-{\text{R}}_{0})/0.5\times 100;\\ \text{Recovery rate }(\%)={\text{R}}_{\text{1}}/0.5\times 100 \end{array}$$

where R_0_ and R_1_ represented the concentrations of OPPs after 24 h incubation with and without *L. plantarum* inoculation, respectively. The initial concentration of the three OPPs was 0.5 mg/kg in MRS culture.

### Extraction of OPPs Residues in MRS Culture

The extraction and purification of OPPs from the MRS culture were performed according to the methods of Pagliuca et al. (2005) with some modifications. Briefly, 20 g of each MRS sample was extracted with 25 mL of acetonitrile-acetone solution (4:1, v/v). The culture-solvent mix was shaken vigorously for 5 min and centrifuged at 4000 ×*g* for 5 min. The supernatant was transferred to a separation funnel, and the pellet was re-extracted with 15 mL of the acetone–acetonitrile mixture as above. The obtained supernatants were combined before the addition of 50 mL of dichloromethane. The mixtures were vigorously shaken for 10 min and left for 30 min for phase separation. The dichloromethane phase was collected and dried by anhydrous sodium sulfate (1.0 g) as a purified pesticide extract. The extracted samples were passed through a 0.22 μm microporous membrane filter before GC-MS analysis.

### Detection of OPPs Residues Using GC-MS

The extracted OPPs were detected in the injector port of a GC-MS (GC-7980-MS-5977, Agilent, United States) equipped with a capillary column (HP-5 Innowax; 30 m × 0.25 mm; ID 0.25 μm film thickness; Agilent, Santa Clara, CA, United States). The carrier gas was helium with a flow of 3 mL/min in splitless mode. The temperature of GC-MS injection port was 100°C. The temperature program was isothermal at 100°C for 1 min, followed by a series of stepwise temperature elevation: 100–195°C at 30°C/min until 195°C for 8 min, 195–202°C at 1°C/min until 202°C for 1 min, 202–205°C at 1°C/min, 205–240°C at 15°C/min, 240–280°C at 8°C/min until 280°C for 10 min, and 280–300°C at 20°C/min until 300°C for 10 min. The MS was operated in the electron impact mode with electron impact energy of 70 eV and ion source temperature of 230°C. Data were collected in single ion monitoring (SIM) at a rate of 0.7 scan/s over a range of m/z 40–400.

To detect the linear detection range of the OPPs and to establish the OPPs standard curves by linear regression analysis, the three pesticide working solutions at appropriate concentration levels (from 0.0625 to 0.500 mg/kg) with equal concentration of each pesticide were mixed accordingly and analyzed with GC-MS. The limit of detection (LOD) of three OPPs was also analyzed with GC-MS by gradient dilution.

### Tolerance of *L. plantarum* Isolates to Bile

The bile tolerance of six selected *L. plantarum* isolates was evaluated (Walker and Gilliland, 1993). Briefly, suspensions of *L. plantarum* (1%, v/v) were added to freshly prepared MRS broth that contained 0.2% (w/v) sodium thioglycollate (Kanto, Japan) and 0.3% (w/v) oxgall (dehydrated fresh bile, DIFCO, Canada). Controls without oxgall were prepared in parallel. The bacterial cultures were incubated at 37°C in a water bath. The absorbance at 600 nm was measured using a spectrophotometer (U-1700, Shimadzu Corporation, Japan) against the corresponding non-inoculated blank. The bacterial growth was followed until a 0.3-unit difference in absorbance was reached; and the lag time was defined as the delay in growth (h) in oxgall-containing culture medium. The experiment was performed in triplicate.

### Tolerance of *L. plantarum* Isolates to Artificial Gastrointestinal Juices

The tolerance of six selected *L. plantarum* to artificial gastrointestinal juices was evaluated (Usman and Hosono, 1999). Briefly, *L. plantarum* isolates were grown in MRS at 37°C overnight. Cells were washed twice with PBS before resuspending in the same buffer. Half milliliter of each bacterial suspension was added to 4.5 mL simulated gastric juice in sterilized PBS (pH 2.5) that contained 0.3% (m/v) pepsin (10,000 U/mg, Sigma-Aldrich, St. Louis, MO, United States). An aliquot of 0.5 mL 3-h incubated gastric juice-bacterial mixture was further inoculated into 4.5 mL simulated intestinal juice that contained 0.1% trypsin (m/v, 2,500 U/mg) and 0.3% bile (m/v) (Sigma-Aldrich, St. Louis, MO, United States, pH 8.0) and incubated at 37°C. The gastric transit tolerance was estimated based on the bacterial survival rate, which was calculated from the total viable counts prior to and 3 h after incubation in simulated gastric juice, as well as after 4 and 8 h of incubation in simulated intestinal juice. The probiotic *L. plantarum* P8 (Bao et al., 2012; Wang et al., 2014) was used as a reference strain in this experiment. The experiment was performed in triplicate.

The survival rate was calculated according to the following equation:

$$\text{Survival rate }(\%)={\text{N}}_{1}/{\text{N}}_{0}\times 100$$

where N_0_ and N_1_ represented the total viable counts of *L. plantarum* before and after incubating in the respective simulated juices.

### Detection of Phosphatase Activity in Samples

The intracellular and extracellular phosphatase activity of six selected *L. plantarum* with high OPPs degradation rate was determined from their culture supernatants and bacterial pellets (Palacios et al., 2005) with some modifications. The bacterial pellets were thawed, resuspended in PBS, and sonicated for 30 min by a Lab ultrasonic cell pulverizer (JY92-H; Ningbo Scientz Biotechnology Co., Ltd., Ningbo, China) at 600 W. The sonicated cell extracts were washed in PBS, and the supernatants were collected. Samples (125 μL) were added to and incubated for 60 min at 37°C with an equal volume of sodium acetate-acetic acid (50 mM, pH 5.0) containing 10 mmol/L *p*-nitrophenyl-phosphate substrate (Fluka Chemie, Buchs, Spain). The reaction was stopped by adding 250 μL of 1 M NaOH. The *p*-nitrophenol released was determined by measuring the absorbance at 405 nm in a multi-well plate spectrophotometer (Synergy^TM^ H1, Microplate Spectrophotometer, Vermont, VT, United States). Standard solutions containing *p*-nitrophenol (ranged 0.125–2 μmol/mL) were used for constructing standard curve in parallel with sample measurement. One unit of phosphatase activity (U) was defined as the amount of enzyme that produced 1 μmol of *p*-nitrophenol per hour at 37°C.

### Effect of Phorate on the Growth of *L. plantarum* P9

To test the effect of phorate on the growth of the strain *L. plantarum* P9, bacteria (1 × 10^7^ cfu/mL) were inoculated into sterile MRS medium containing four different concentrations of phorate, i.e., 0, 0.5, 2.5, and 10 mg/kg. The inoculated cultures were incubated at 37°C, and the viable counts and OD_600_ value were determined at 0, 2, 4, 6, 8, 12, 16, 20, and 24 h of incubation.

### Metabolomic Analysis of Phorate Degradation by *L. plantarum* P9

#### Sample Preparation

The metabolites of phorate degradation by *L. plantarum* P9 were detected based on the method of Lénárt et al. (2013) with some modifications. Briefly, the strain P9 was allowed to grow in MRS medium with (10 mg/kg) or without phorate at 37°C for 24 h. After incubation, the phorate residues were extracted as described above. Then, the phorate residues were further extracted using the Waters^®^ Ossasis Vac C18 3cc disposable cartridges (Waters, Milford, MA, United States). The cartridges were first activated with 2 mL of methanol and 2 mL of water. Afterward, 2 mL of each sample was loaded onto an activated cartridge. Impurities were washed with 3 mL of water. Then, 3 mL of acetonitrile was used to elute the sample. The eluent was collected and filtered through a disposable 0.22 μm syringe filter before analysis with UPLC/ESIQ-TOF/MS.

#### Conditions for UPLC/ESI-Q-TOF/MS

The UPLC/ESI-Q-TOF/MS (Waters United States) was equipped with ACQUITY UPLC BEH C18 column (100 mm × 2.1 mm, 1.7 μm, Waters, United States). The UPLC flow rate was set to 300 μL/min. For the chromatographic gradient, solution A was 0.1% (v/v) formic acid, while solution B was acetonitrile. Gradient elution was set as follows: 0–10 min 10% B; 10–18 min up to 50% B; 18–20 min 95% B, 20-21 min down to 10% B. The Q-TOF-MS instrument was operated with dual ESI ion source in positive and negative ionization modes. Mass calibration was performed by introducing a mass calibration solution by direct infusion according to the manufacturer’s instructions. This solution contains internal reference masses [Leu]enkephalin (C_28_H_37_N_5_O_7_). Afterward, the eluent was analyzed by a high definition mass spectrometer (Waters, United States) under the following conditions: the mass spectrometry was in positive ionization mode; the source temperature was 100°C; the desolvation gas temperature was 350°C; the cone gas flow was 50 L/h; the desolvation gas flow was 800 L/h; the capillary voltage was 3.0 kV; the sampling cone voltage was 40.0 V; and the extraction cone voltage was 4.1 V. The mass spectrometric data were collected over the range of 100–800 (m/z).

#### Identification of Differential Abundant Metabolites

All UPLC/ESI-Q-TOF/MS collected data were analyzed by the Masslynx Application Manager software (V4.1, Waters, United States). Specifically, the fragment ion data were extracted and analyzed by the chemically intelligent peak-matching algorithms method. Then, the count of each ion was normalized with the total ion count to generate a data matrix consisting of the retention time, m/z value, and normalized peak area (10,000). The multivariate data matrix was analyzed by the EZinfo software 2.8 (Waters, United States). Finally, all the variables were mean-centered and Pareto-scaled before orthogonal partial least squares discriminant analysis (OPLS-DA). Differential abundant metabolites were those having a VIP value of ≥1. The metabolites of interest were identified and confirmed by comparing their mass spectra with the information available in open biochemical databases, such as METLIIN^2^, HMDB^3^, KEGG^4^, and MASSBANK^5^.

### Data and Statistical Analyses

All data were collected from three independent sets of experiments; data were expressed as mean ± standard deviation (SD). Statistically significant differences between sample groups were evaluated with analysis of variance (ANOVA). Pearson correlation analysis and ANOVA were performed with the SPSS software (version 13, SPSS/IBM, Chicago, IL, United States). *P* < 0.05 was considered as statistically significant. The software, OriginPro 2015 (OriginLab Corporation, Northampton, MA, United States), was used to plot graphs. The metabolomic data sets were then imported into SIMCA-P version 12 (Umetrics, Umeå, Sweden) for OPLS-DA, PCA, and permutation test (seven-round cross-validation).

## Results

### Initial Screening for OPPs Degrading *L. plantarum* Strains

**FIGURE 1:**
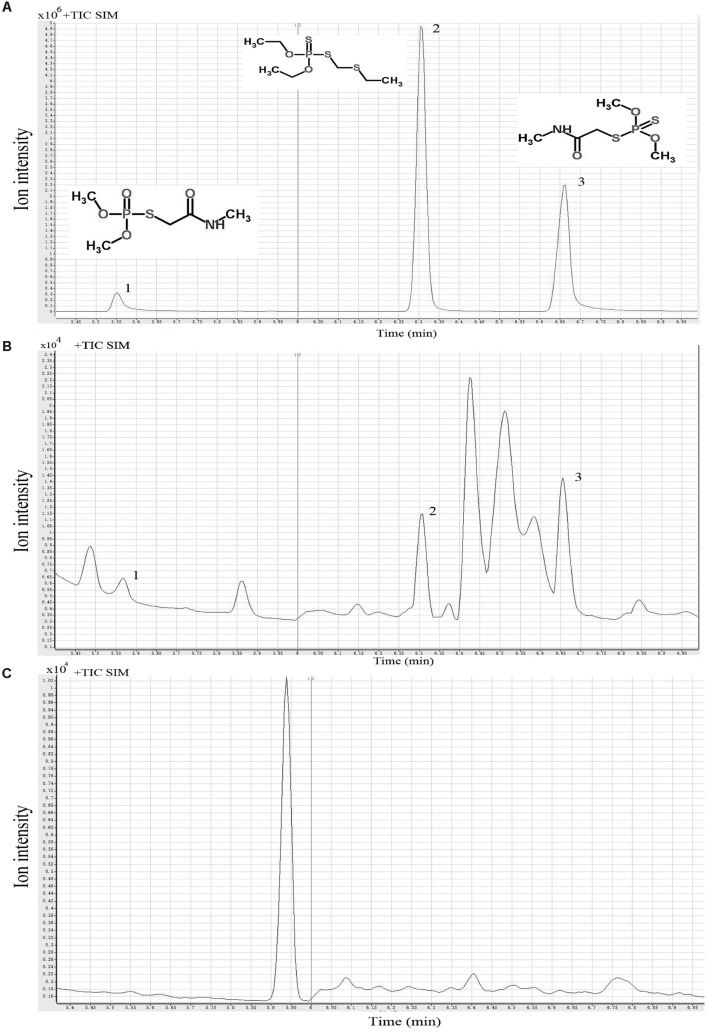
Typical single ion monitoring (SIM) gas chromatography-mass spectrometry (GC-MS) profiles of three organophosphorus pesticides. Standard solution **(A)**, culture supernatant **(B)**, and sonicated cell extract **(C)**. Peaks 1-3 represent omethoate, phorate, and dimethoate, respectively.

The OPPs degrading capacity of 121 *L. plantarum* strains was screened by GC-MS analysis of dimethoate, phorate, and omethoate residues in the culture medium of *L. plantarum* cultures. Typical GC-MS profiles and chemical structures of the mixture of the standard solutions of these three OPPs, culture supernatant, and sonicated cell extract are given in **Figures 1A–C**, respectively. The three pesticides appeared as three distinct peaks on the chromatograph using the current experimental conditions (**Figure 1A**). The standard curves of the three OPPs showed excellent linear reliability, with *R*^2^ value ranged from 0.978 to 0.995, spanning the concentration from 0.0625 to 0.500 mg/kg (**Table 1**). The limit of detection (LOD) for the three pesticides ranged from 0.006 to 0.012 mg/kg; and the percentage recovery of dimethoate, phorate, and omethoate was 112.42 ± 1.64%, 107.30 ± 10.88%, and 89.78 ± 1.98%, respectively (**Table 1**). These results verified that the current extraction, purification, and detection procedures were suitable for analyzing the pesticide residues in our samples.

**Table 1:** The retention time, recovery, and limit of detection (LOD) of the 3 organophosphorus pesticides detected by gas chromatography mass spectrometry (GC-MS).

	Molecular formula	Average mass (Da)	Retention time (min)	*R*^2^	Recovery (%)	LOD (mg/kg)
Omethoate	C_5_H_12_NO_4_PS	213.192	5.55	0.978	89.78 ± 1.98	0.012
Phorate	C_7_H_17_O_2_PS_3_	260.377	6.32	0.995	107.30 ± 10.88	0.006
Dimethoate	C_5_H_12_NO_3_PS_2_	229.257	6.66	0.991	112.42 ± 1.64	0.012

*The recovery rate of each organophosphorus pesticide was determined at a fixed concentration of 0.5 mg/kg. ‘R^2^’ is the coefficient of determination. ‘LOD’ is the limit of detection for each organophosphorus pesticide as detected by GC-MS.*

The capacity of OPPs degradation of the 121 food-derived *L. plantarum* isolates is shown (**Supplementary Table 1**). Most of the screened *L. plantarum* strains showed some levels of degradation activity against omethoate, phorate, and dimethoate, respectively. The percentage degradation for omethoate, phorate, and dimethoate ranged from 1.99 ± 0.96% to 13.57 ± 0.14%, 15.52 ± 7.67 to 36.29 ± 0.04%, and 2.85 ± 0.00% to 27.32 ± 2.23%, respectively. The screening criteria for potential OPPs degrading *L. plantarum* were degradation rates of >9%, 30%, and 9% for dimethoate, phorate, and omethoate, respectively. Based on these results, six strains (P9, IMAU80110, IMAU40100, IMAU10585, IMAU10209, and IMAU80070) that showed good OPPs degradation ability were selected for further study (**Table 2**).

**Table 2:** Degradation of organophosphorus pesticides by 6 selected *Lactobacillus plantarum* strains.

Strain No.	NCBI accession number	Food source of the bacterial strain	Sampling region	Degradation of organophosphorus pesticides		
				Omethoate (%)	Phorate (%)	Dimethoate (%)
*Lactobacillus plantarum* P9	GQ131126	Sour porridge	Inner Mongolia	11.37 ± 1.42^b^	35.52 ± 0.50^a^	14.00 ± 2.97^c^
IMAU10585	HM218309	Koumiss	Inner Mongolia	10.95 ± 0.95^b,c^	36.02 ± 0.87^a^	16.36 ± 0.59^c^
IMAU10209	GU138537	Sour dough	Inner Mongolia	9.88 ± 0.21^c,d^	32.86 ± 0.70^b^	27.32 ± 2.23^a^
IMAU40100	FJ749375	Koumiss	Qinghai	13.57 ± 0.14^a^	31.16 ± 1.49^c^	9.01 ± 3.15^d^
IMAU80070	GU125492	Pickle	Sichuan	9.05 ± 0.20^d^	32.41 ± 0.36^b,c^	22.76 ± 1.32^b^
IMAU80110	GU125532	Pickle	Sichuan	13.68 ± 0.07^a^	33.78 ± 1.06^b^	9.42 ± 2.13^d^

*Data are expressed in mean ± SD. The degradation rate of different strains with different letters shown significant difference (*P* < 0.05).*

### Tolerance of the Selected *L. plantarum* to Simulated Gastrointestinal Juices and Bile

To explore the potential of using these isolates in human, their tolerance to simulated gastrointestinal juices and bile was determined (**Table 3**). The survival rates of P9 in simulated gastric juice for 3 h (pH 2.5), simulated intestinal juice for 4 h (pH 8.0), and simulated intestinal juice 8 h (pH 8.0) were 62.74 ± 0.82%, 93.16 ± 7.85%, and 89.08 ± 4.94%, respectively. The lag time of *L. plantarum* P9 grown in bile-containing medium was 1.91 ± 0.11 h. The strain P9 showed good tolerance toward the simulated gastrointestinal juices and bile, and it was thus selected for further metabolomic analysis for phorate degradation.

**Table 3:** Tolerance of 7 selected *Lactobacillus plantarum* isolates to simulated gastrointestinal juices and bile.

Isolates	Survival in simulated gastric juice at pH 2.5			Survival in simulated intestinal juice at pH 8				Survival in bile		
	0 h (× 10^8^ cfu/mL)	3 h^#^ ( × 10^7^ cfu/mL)	Survival after 3 h (%)	4 h ( × 10^6^ cfu/mL)	Survival after 4 h^∗^ (%)	8 h ( × 10^6^ cfu/mL)	Survival after 8 h^∗^ (%)	No oxgall (OD _620_)	0.3% (w/v) oxgall (OD _620_)	Lag time (h)
P8	0.47 ± 0.07	1.85 ± 0.30	39.64 ± 1.07^d^	2.18 ± 0.36	118.02 ± 9.95^d^	1.85 ± 0.13	99.91 ± 5.44^d^	3.30 ± 0.05	4.38 ± 0.08	1.08 ± 0.08^c^
P9	0.91 ± 0.06	5.73 ± 0.23	62.74 ± 0.82^e^	5.33 ± 0.78	93.16 ± 7.85^c^	5.10 ± 0.37	89.08 ± 4.94^d^	2.96 ± 0.11	4.87 ± 0.11	1.91 ± 0.11^d^
IMAU10585	0.86 ± 0.16	1.78 ± 0.18	19.90 ± 1.52^c^	2.25 ± 0.22	126.76 ± 7.97^d^	2.13 ± 0.19	119.72 ± 9.86^e^	3.60 ± 0.17	3.97 ± 0.03	0.37 ± 0.03^a^
IMAU40100	2.10 ± 0.17	3.20 ± 0.47	15.25 ± 2.06^b^	1.75 ± 0.15	54.53 ± 3.13^b^	2.12 ± 0.34	66.30 ± 8.37^c^	3.35 ± 0.02	5.63 ± 0.17	2.29 ± 0.17^e^
IMAU80070	3.50 ± 0.37	1.84 ± 0.15	5.36 ± 0.09^a^	1.66 ± 0.11	90.22 ± 3.53^c^	1.76 ± 0.13	95.83 ± 6.62^d^	3.43 ± 0.05	5.47 ± 0.06	2.04 ± 0.06^d^
IMAU80110	2.58 ± 0.78	1.53 ± 0.09	5.95 ± 0.34^a^	0.90 ± 0.28	58.84 ± 18.16^b^	0.85 ± 0.01	55.27 ± 0.65^b^	2.59 ± 0.06	5.81 ± 0.06	3.22 ± 0.06^f^
IMAU10209	1.65 ± 0.04	6.84 ± 0.11	41.42 ± 0.64^d^	2.54 ± 0.18	37.19 ± 2.63^a^	2.57 ± 0.05	37.54 ± 0.79^a^	3.61 ± 0.05	4.41 ± 0.10	0.81 ± 0.10^b^

*^#^Number of surviving cells after 3 h incubation in gastric juice. ^∗^The survival in simulated intestinal juice was calculated by comparing the number of surviving cells after incubation for 4 or 8 h in simulated intestinal juice with the number of surviving cells after 3 h incubation in gastric juice. Data are expressed as means of triplicate replicates ± standard deviation. Different superscript letters represent significant differences of different strains under the same treatment (P < 0.05).*

### Acid Phosphatase Activity of *L. plantarum*

**FIGURE 2:**
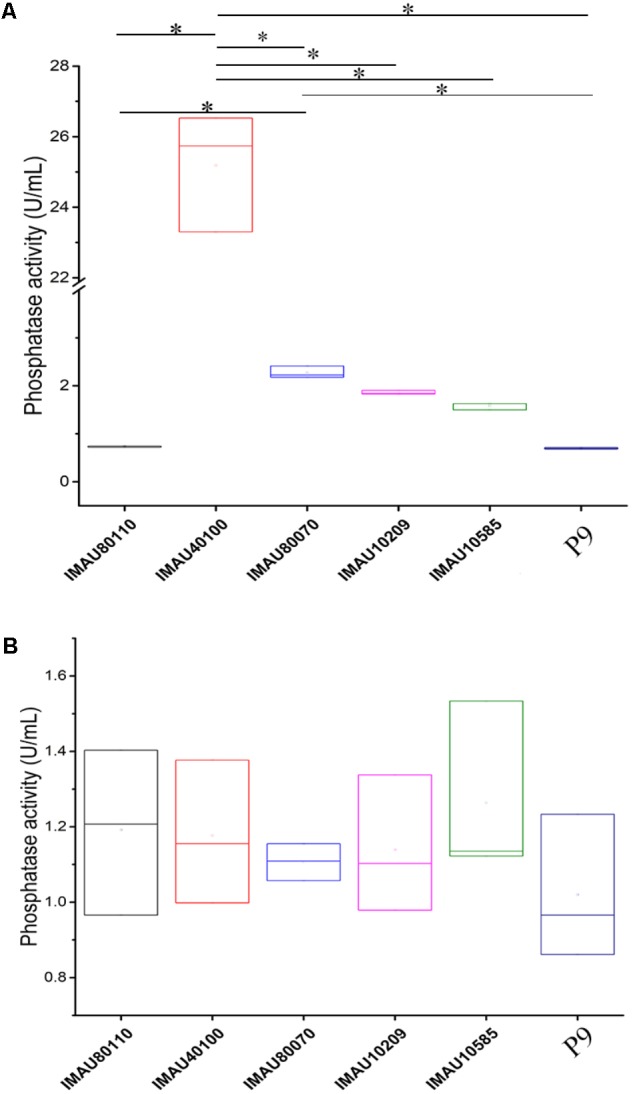
The intracellular **(A)** and extracellular **(B)** phosphatase activities of six selected *Lactobacillus plantarum* strains grown in organophosphorus pesticides-containing culture medium. Significant differences in the extracellular phosphatase activity between strains are indicated (^∗^*P* < 0.05), while no significant difference was detected in the intracellular phosphatase activity.

The intracellular and extracellular acid phosphatase activities of the six selected *L. plantarum* strains are shown in **Figure 2**. The intracellular and extracellular phosphatase activity of these bacteria ranged from 0.69 ± 0.02 to 25.19 ± 1.68 U/mL and 1.02 ± 0.19 to 1.26 ± 0.23 U/mL, respectively. However, no significant correlation (*P* > 0.05) existed between the OPPs degradation rates and the intracellular and extracellular acid phosphatase activities (**Supplementary Table 2**). It was interesting to note that the extracellular phosphatase activity of *L. plantarum* IMAU40100 (25.19 ± 1.68 U/mL) was significantly higher than that of other strains (*P* < 0.05).

### Effect of Phorate on the Growth of *L. plantarum* P9

**FIGURE 3:**
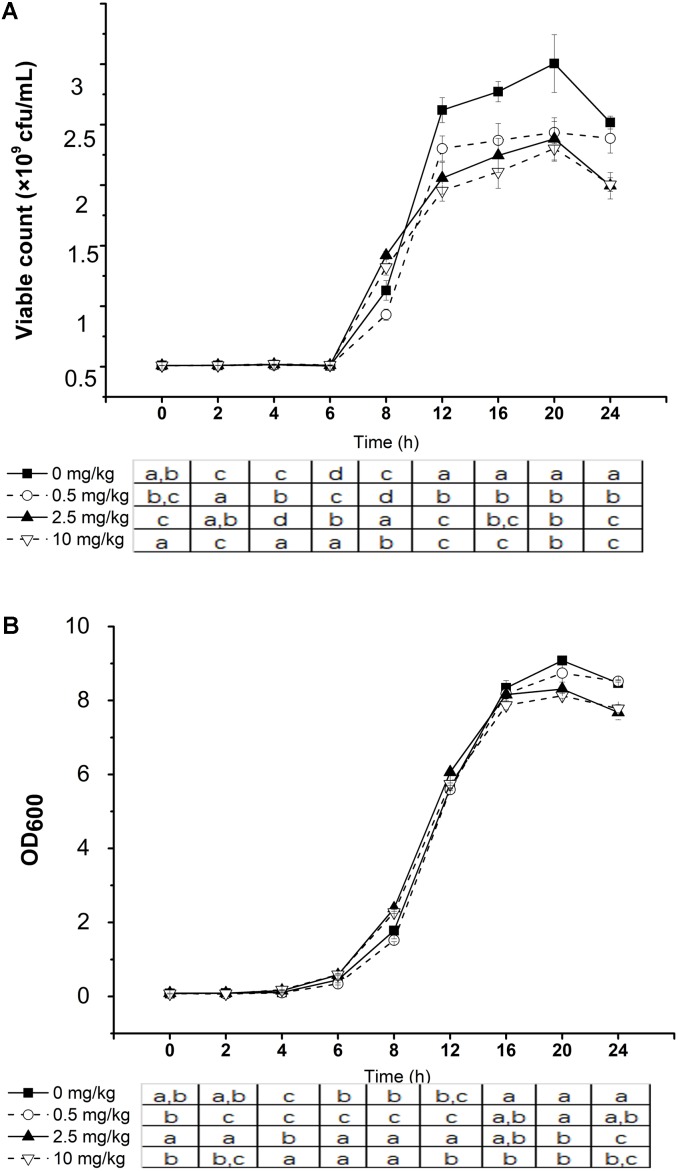
Effect of phorate concentration on the growth of *Lactobacillus plantarum* P9. Viable count **(A)** and optical density at 600 nm **(B)** in the same fermentation. Significant differences between pesticide concentration used are shown by different letters (^∗^*P* < 0.05). Error bars represent standard deviation.

Changes in the viable counts (**Figure 3A**) and OD_600_ value (**Figure 3B**) of *L. plantarum* P9 culture with and without phorate addition were monitored. The bacteria grew significantly slower in the presence of phorate only after 12 h of incubation (*P* < 0.05). Furthermore, *L. plantarum* P9 cultured in phorate-containing medium (only 2.5 and 10 mg/kg) showed significantly lower viable counts and OD_600_ value compared with the control.

Although the bacterial growth was slower in the presence of phorate, changes in the metabolic profiles reflected pesticide utilization and degradation. After 24 h of incubation, the relative abundances of citric acid (increased 6.98 and 634.57 folds in positive and negative ion modes, respectively), malic acid (increased 11.71 folds), and succinic acid (increase 12.46 folds) were significant higher in culture containing a high phorate concentration (10 mg/kg) compared with the control without phorate (*P* < 0.05, fold change ≥ 2 in all cases) (**Table 4**).

**Table 4:** Potential phorate-specific differential abundant markers of *Lactobacillus plantarum* P9.

No.^#^	Molecular name	Molecular formula	Retention time	m/z	Fold change^∗^	VIP value	Metabolic pathways
P1	Dimethyl hexadecylphosphonate	C1_8_H_39_O_3_P	19.99	335.2805	3676.1000	1.17	–
P2	Sphinganin	C_18_H_39_NO_2_	12.07	302.3056	5.9112	3.36	Glycosphingolipid metabolism
P3	5,8-Icosadienoic acid	C_20_H_36_O_2_	18.24	309.2796	4.8346	5.13	–
P4	4,4-Dimethoxy-2,5-cyclohexadien-1-one	C_8_H_10_O_3_	19.20	155.1070	7547.5000	1.63	–
P5	Citric acid	C_6_H_8_O_7_	8.89	193.0501	6.9857	1.52	Citric acid cycle
P6	Malic acid	C_4_H_6_O_5_	12.40	135.0795	11.7050	2.26	Citric acid cycle
P7	*O,O,O*-Tris(2-methylphenyl) thiophosphate	C_21_H_21_O_3_PS	8.94	385.0913	6.3126	2.28	–
P8	Succinic acid	C_4_H_6_O_4_	12.40	119.0836	12.4630	3.14	Citric acid cycle
P9	11-Mercaptoundecanol	C_11_H_24_OS	19.10	205.1967	3775.3000	1.14	–
P10	Alanylphenylalanine	C_12_H_16_N_2_O_3_	8.95	237.0767	8.3566	1.45	–
P11	(2,2-Diethoxyethoxy)benzene	C_12_H_18_O_3_	19.28	211.1573	3781.5000	1.12	–
P12	*N,N*-Dimethyl-*N*-(3-sulfopropyl)-1-tetradecanaminium	C_19_H_42_NO_3_S	18.99	366.3727	6323.6000	1.44	–
P13	18-Pentatriacontanyl Dihydrogen phosphate	C_35_H_73_O_4_P	18.12	589.5542	7572.3000	1.57	–
P14	5,8-Icosadienoic acid	C_20_H_36_O_2_	16.53	309.2791	5805.5000	1.37	–
P15	Tripentylphosphine oxide	C_15_H_33_OP	18.23	261.2585	3517.4000	1.06	–
P16	Oleic (*cis*-9-octadecenoic) acid	C_18_H_34_O_2_	18.77	283.2645	<0.0001	2.01	Fatty acid biosynthesis
P17	*N*-Pentadecanoyl-L-tyrosine	C_24_H_39_NO_4_	19.56	406.3284	0.2866	4.28	–
P18	*N*-(2-Hydroxyethyl)-5,8,11,14-icosatetraenamide	C_22_H_37_NO_2_	18.23	348.3065	0.0003	1.01	–
P19	2-Isopropylmalate	C_7_H_12_O_5_	19.09	177.113	0.0002	1.35	Biosynthesis of amino acids
P20	9,12-Dioxooctadecanoic acid	C_18_H_32_O_4_	19.45	313.2729	0.0002	1.27	–
P21	L-Isoleucyl-L-lysyl-L-valyl-L-alanyl-L-valine	C_25_H_48_N_6_O_6_	20.71	529.4089	0.0002	1.30	–
P22	2,4,6-Octatrienoic acid	C_8_H_10_O_2_	18.78	139.1106	0.0001	1.70	–
N1	Diphosphate	O_7_P_2_	3.61	172.9489	613.3600	1.02	Citric acid cycle/Purine metabolism
N2	Citric acid	C_6_H_8_O_7_	5.46	191.0525	634.5700	1.02	Citric acid cycle
N3	Phosphocreatine	C_5_H_11_N_2_O_5_P	6.28	209.0636	3406.0000	2.42	Arginine and proline metabolism
N4	2-Phosphoglycolic acid	C_2_H_5_O_6_P	7.03	154.9448	623.5100	1.09	–
N5	(2S)-2-Hydroxy-3-(phosphonooxy)propyl hexanoate	C_9_H_19_O_7_P	13.01	269.0324	8.3203	1.94	–
N7	1-Palmitoyl lysophosphatidic acid	C_19_H_39_O_7_P	16.69	409.2444	699.9600	1.08	–
N8	Phosphonoacetaldehyde	C_2_H_5_O_4_P	14.70	122.9873	0.0016	1.05	Phosphonate and phosphinate metabolism
N9	Cystine	C_6_H_12_N_2_O_4_S_2_	20.90	239.0562	0.0019	1.00	Cysteine and methionine metabolism

*^#^P1-P22 and N1-N2 represent the compounds identified by positive and negative ion modes, respectively. ^∗^The fold change value is the ratio of signal intensity between cells grown with and without phorate.*

*“–” Means that the identified compound does not belong to any known metabolic pathways.*

### Metabolites of Phorate Degradation Released by *L. plantarum* P9

Phorate potential degradative metabolites were detected by OPLS-DA (**Table 4**). The robustness of the OPLS-DA based on metabolite profiles generated in positive and negative ion modes was assessed by a permutation test (200 repetitions) (**Supplementary Figures 2a,b**). The *R*^2^ and *Q*^2^ values were (0.999, 0.759) and (1.000, 0.975) in positive and negative modes, respectively; and *R*^2^ and *Q*^2^ values derived from the permuted data were lower than that of the original ones at the right side of the plots. In general, *R*^2^ measures the goodness of fit, while *Q*^2^ measures the predictive ability of the model; An *R*^2^ close to 1 indicates perfect description of the data by the model, whereas an *Q*^2^ close to 1 indicates perfect predictability (Triba et al., 2015). Moreover, *Q*^2^ > 0.5 is considered good predictability according to the SIMCA-P12 users’ guide. Thus, the permutation results supported a high validity of the current OPLS-DA models.

The detected phorate degradative metabolites released by *L. plantarum* P9 are listed in **Table 4**. Many of the identified metabolites contained phosphate or phosphorus atoms. For example, dimethyl hexadecylphosphonate (3676.10-fold), *O,O,O*-Tris(2-methylphenyl) thiophosphate (6.31-fold), 18-pentatriacontanyl dihydrogen phosphate (7572.30-fold), and tripentylphosphine oxide (3517.40-fold), were detected in positive ion mode, while diphosphate (613.36-fold), phosphocreatine (3406.00-fold), 2-phosphoglycolic acid (623.51-fold), (2S)-2-Hydroxy-3-(phosphonooxy)propyl hexanoate (8.32-fold), phosphonoacetaldehyde (0.00-fold), and 1-Palmitoyl lysophosphatidic acid (699.96-fold) were detected in negative ion mode.

## Discussion

Our study screened 121 food-originated *L. plantarum* strains for their capacity of degrading dimethoate, phorate, and omethoate; and our results showed that their degradation activities toward the three OPPs were obviously different. The most biodegradable pesticide was phorate (dissipation rate ranging from 14.22 ± 7.88% to 36.29 ± 0.04%), which was generally higher than that of omethoate and dimethoate. Similar observations were reported in another study showing phorate was the most degradable OPPs among 9 different pesticides by different lactic acid bacteria (Zhou and Zhao, 2015). This may due to the low energy P-S and C-S bonds present in phorate (Wu et al., 2010). The pesticide-degrading ability of *Lactobacillus* has previously been demonstrated. For example, *L. plantarum* could enhance degradation of pirimiphos-methyl during wheat fermentation (Đorđević et al., 2013); *L. plantarum* WCP931 could degrade chlorpyrifos in kimchi fermentation (Cho et al., 2009); some *Lactobacillus* spp. could degrade up to seven OPPs in skimmed milk culture (Zhao and Wang, 2012; Zhou and Zhao, 2015). Our study found that the 121 *L. plantarum* strains could variably degrade the three investigated OPPs; and six of the strains (P9, IMAU10585, IMAU10209, IMAU40100, IMAU80070 and IMAU80110) showed good capacity of degrading all three tested pesticides. Thus, they were selected for further characterization.

Although some lactobacilli could potentially reduce unavoidable pesticide absorption in humans (Trinder et al., 2015), no report has explored their survival in the harsh environments through gastrointestinal transit, e.g., a strong acidity in the stomach, a high level of digestive enzyme (like pepsin and chymotrypsin) activities and bile salts present in gastrointestinal juices (De Vries et al., 2006). Thus, we tested the tolerance of six selected OPPs-degrading strains to artificial gastrointestinal juices and bile (**Table 3**). The *L. plantarum* P8 strain (IMAU10120) was used as the control in this experiment. It is a well-characterized strain that has high gastrointestinal transit survival and confers numerous desirable effects to the host (Bao et al., 2012; Wang et al., 2014). Among the six selected OPPs-degrading strains, P9 matched most to the gastrointestinal tolerance property of *L. plantarum* P8, with a high survival in simulated gastric juice of pH 2.5 for 3 h (62.74 ± 0.82%), intestinal juice for 4 h (93.16 ± 7.85%) and 8 h (89.08 ± 4.94%). Such properties are crucial criteria to consider when probiotics-based products are to be developed for use in human and/or animals. In our body, OPPs are mainly absorbed through the gastrointestinal tract and transported to the liver, the major site of pesticide metabolism, via the blood circulation; and it increases the risk in liver toxicity (Harishankar et al., 2013). Therefore, the gastrointestinal tract is the major target site for pesticide degradation. The *L. plantarum* P9 strain exhibited high potential of gastrointestinal transit survival based on its good tolerance to the simulated gastrointestinal juices. Moreover, *L. plantarum* P9 was able to colonize the rat intestine; it could lower rat phosphorus poisoning by excreting organic phosphorus pesticides and reduce gut inflammation (unpublished data).

Generally, pesticide dissipation is either via degradation (Sharma et al., 2005; Lénárt et al., 2013) or absorption (Ruediger et al., 2005; Uygun et al., 2008). Our GC-MS analysis detected residue dimethoate, phorate, and omethoate mainly in the culture supernatants but not the cell extracts, suggesting that the strain P9 degraded rather than absorbed the OPPs. Several groups of microbial enzymes, including carboxylesterases, phosphatases (Palacios et al., 2005; Bhalerao and Puranik, 2009), phosphotriesterases (Buratti and Testai, 2005; Weston and Amweg, 2007), organophosphorus hydrolases (Islam et al., 2010), may facilitate OPPs degradation via the hydrolysis of phosphoric acid esters. Both acid and alkaline phosphatases may degrade OPPs by hydrolyzing the C-O-P linkage of a wide variety of phosphate esters (Kadam et al., 2003; Palacios et al., 2005; Thengodkar and Sivakami, 2010; Zhang et al., 2014). Zhang et al. (2014) reported strong positive correlation between fermented milk phosphatase activities and OPPs degradation (*r* = 0.636–0.970, *P* < 0.05). Zhou and Zhao (2015) also observed correlation between phosphatase production of *L. bulgaricus* and OPP dissipation. However, our work did not find such correlation (P > 0.05), suggesting that the current pesticide-degrading mechanism might be relating to enzymes other than acid phosphatase.

Stress may be defined as modification of the growth environment that leads to an adverse cell response (Thammavongs et al., 2008). The reduction of growth of *L. plantarum* P9 in the presence of the xenobiotic, phorate, suggests that this chemical induced stress to the bacteria. The inhibition of bacterial growth has been observed with the use of other pesticides. For instance, Harishankar et al. (2013) reported low survival and growth rates of gut-originated *L. plantarum* in chlorpyrifos (100 μg/mL), particularly in comparison to *Lactococcus lactis* (1,500 μg/mL) and *L. fermentum* (1,500 μg/mL). Clair et al. (2012) demonstrated a suppressive effect of glyphosate on *Lactococcus lactis* subsp. *cremoris* and *L. delbrueckii* subsp. *bulgaricus*. Similarly, Joly et al. (2013) reported diminished survival of *Lactobacillus* and *Bifidobacterium* in simulated intestine juices in the presence of chlorpyrifos. Bacteria vary greatly in their physiological and cellular responses in adapting to environmental stress.

By metabolomic analysis, apparent alterations of the metabolome profiles were observed in cell culture supernatants that contained phorate in comparison with the non-pesticide containing ones. Significant increases were observed in sphinganin (5.91-fold), citric acid (6.99-fold in positive mode; 634.57-fold in negative mode), malic acid (11.71-fold), and succinic acid (12.46-fold) in the phorate-containing culture (*P* < 0.05). Citric acid, malic acid, and succinic acid were the intermediates of the tricarboxylic acid (TCA). However, the genome of *L. plantarum* P9 does not code for the complete TCA cycle (**Supplementary Figure 1**). Thus, it was unlikely that the excessive citric acid, malic acid, and succinic acid detected in the culture supernatant were produced by P9. Indeed, citric acid and malic acid could be used by *L. plantarum* for its growth and eventually metabolized to form acetic acid and lactic acid, respectively (Nualkaekul and Charalampopoulos, 2011; Das et al., 2017). The high level of these metabolites could indeed be explained by the reduced growth of bacterial cells, possibly via blockage of related metabolic pathways by phorate, leading to the accumulation of these TCA intermediates in the culture medium.

The other reason for the alteration of metabolomic profiles of the culture medium could be related to phorate degradation and release of degradation residues in the culture medium. Although many studies have demonstrated the ability of lactic acid bacteria in degrading OPPs (Zhao and Wang, 2012; Đorđević et al., 2013; Zhang et al., 2016), the detailed mechanisms have not been characterized with limited reports focusing on analyzing the profile of decomposed metabolites. Phorate is thioester of phosphoric acid with a central phosphorus atom. The potential metabolites released upon microbial phorate hydrolysis include diethyl dithiophosphate, triethyl dithio-phosphate, diethyl disulfide, formaldehyde, and hydrogen sulfide (Singh et al., 2003; Rani et al., 2009; Jariyal et al., 2014). Our work identified a number of potential phorate-derived metabolites released by cultivation of *L. plantarum* in phorate-containing MRS. These included methyl ether bond compounds (e.g., dimethyl hexadecylphosphonate, 4,4-dimethoxy-2,5-cyclohexadien-1-one, and tripentylphosphine oxide), -P = S- bond compound [e.g., *O,O,O*-Tris(2-methylphenyl) thiophosphate], and phosphoric acid group compounds (e.g., 18-Pentatriacontanyl dihydrogen phosphate, diphosphate, phosphocreatine, 2-Phosphoglycolic acid, (2S)-2-Hydroxy-3-(phosphonooxy)propyl hexanoate, 1-palmitoyl lysophosphatidic acid, and phosphonoacetaldehyde) (**Table 4**).

The phosphoric acid group metabolic compounds are similar to the diethyl phosphoric acid in chemical structure, which have been reported as phorate degradation products released by *Azotobacter* (Kadam and Gangawane, 2005). Among the metabolites identified in our study, *O,O,O*-Tris(2-methylphenyl) thiophosphate with P = S bond is similar to diethyl dithiophosphate, which has been reported as a phorate degradation metabolite produced by *Ralstonia eutropha* (Rani et al., 2009). The decomposed metabolites of phorate seem to be derived from the oxidizing attack of hydroxyl radical and the substitution of sulfur by oxygen in the P-S bond (Wu et al., 2010). Although some of the phorate degradation metabolites detected in our work have previously been reported in literature, further work will be required to elucidate the detailed mechanism of phorate dissipation by microorganisms, particularly the enzymes and metabolic pathways involved in the degradation process.

## Conclusion

This work analyzed the OPPs degradation capacity of 121 food-originated *L. plantarum* isolates. Most of the investigated bacteria displayed some pesticide-degrading activity. Among them, the strain *L. plantarum* P9 was selected because of its high OPPs degradation efficiency for all three investigated OPPs, as well as its high tolerance to simulated gastrointestinal juices and bile. Our results suggested that the OPPs degradation of *L. plantarum* was not responsible by acidic phosphatase. Finally, by metabolomic analysis, we identified a number of differential abundant metabolites when *L. plantarum* P9 was grown in the presence of phorate. These might be potential degradation products released during the process of phorate dissipation.

## Author Contributions

HZ and YC conceived and designed the experiments. CL, YM, RH, TZ, and HH performed the experiments. CL, LK, and ZM analyzed the data. CL, YM, HR, TZ, ZS, and HH contributed reagents, materials, and analysis tools. CL, LK, and YC wrote the paper.

## Conflict of Interest Statement

The authors declare that the research was conducted in the absence of any commercial or financial relationships that could be construed as a potential conflict of interest. The reviewer WL and handling Editor declared their shared affiliation.

## Footnotes

1 http://npic.orst.edu/

2 http://metlin.scripps.edu/

3 http://www.hmdb.ca/

4 http://www.genome.jp/kegg/

5 http://www.massbank.jp/
