# Supplementary material for: Screening for Lactobacillus plantarum Strains That Possess Organophosphorus Pesticide-Degrading Activity and Metabolomic Analysis of Phorate Degradation
Source: Front Microbiol. 2018 Sep 3;9:2048. doi: 10.3389/fmicb.2018.02048 (PMC6130228; doi:10.3389/fmicb.2018.02048)
Supplement: Supplementary file 1 [file Table_1.docx]

**Supplement Table 1** Degradation of organophosphorus pesticides by 121 *Lactobacillus plantarum* strains

| Strain No. | NCBI accession number | Food source of the bacterial strain | Sampling region | Degradation rates of organophosphorus pesticides (%) | | |
| --- | --- | --- | --- | --- | --- | --- |
|  |  |  |  | Omethoate | Phorate | Dimethoate |
| *Lactobacillus plantarum* P9 | GQ131126 | Sour porridge | Inner Mongolia | 11.37±1.42 | 35.52±0.50 | 14.00±2.97 |
| *Lactobacillus plantarum* P8 | FJ915776 | Yoghurt | Inner Mongolia | 4.21±1.28 | 31.40±2.90 | 10.57±3.80 |
| IMAU10585 | HM218309 | Koumiss | Inner Mongolia | 10.95±0.95 | 36.02±0.87 | 16.36±0.59 |
| IMAU80182 | GU125602 | Pickle | Sichuan | 7.04±3.02 | 35.00±0.83 | 15.06±0.15 |
| IMAU80087 | GU125509 | Pickle | Sichuan | 4.38±0.28 | 29.33±1.15 | 6.75±2.08 |
| IMAU10216 | GU138544 | Sour dough | Inner Mongolia | 8.69±0.97 | 32.35±0.41 | 8.26±1.78 |
| IMAU80149 | GU125571 | Pickle | Sichuan | 7.52±2.42 | 31.10±1.13 | 11.63±4.72 |
| IMAU20063 | FJ640996 | Fermented camel milk | Mongolia | 7.55±0.14 | 29.32±0.73 | 11.40±1.97 |
| IMAU10140 | FJ915796 | Yoghurt | Inner Mongolia | - | 25.24±0.26 | 8.93±2.48 |
| IMAU20118 | HM057856 | Yoghurt | Mongolia | 5.35±1.92 | 28.91±2.22 | 10.82±1.27 |
| IMAU80185 | GU125605 | Pickle | Sichuan | 10.56±0.39 | 33.83±0.74 | 8.29±1.01 |
| IMAU80110 | GU125532 | Pickle | Sichuan | 13.68±0.07 | 33.78±1.06 | 9.42±2.13 |
| IMAU80005 | GU125427 | Pickle | Sichuan | 9.52±0.62 | 31.55±1.38 | 8.07±2.41 |
| IMAU40100 | FJ749375 | Koumiss | Qinghai | 13.57±0.14 | 31.16±1.49 | 9.01±3.15 |
| IMAU10209 | GU138537 | Sour dough | Inner Mongolia | 9.88±0.21 | 32.86±0.70 | 27.32±2.23 |
| IMAU80177 | GU125597 | Pickle | Sichuan | 1.99±0.96 | 35.54±1.19 | - |
| IMAU80105 | GU125527 | Pickle | Sichuan | 3.16±0.63 | 34.61±0.84 | - |
| IMAU80173 | GU125593 | Pickle | Sichuan | 6.99±0.31 | 35.12±0.29 | - |
| IMAU80169 | GU125589 | Pickle | Sichuan | 4.44±1.35 | 35.84±0.54 | 2.12±1.49 |
| IMAU80174 | GU125594 | Pickle | Sichuan | 7.91±1.65 | 34.77±2.49 | 5.01±1.03 |
| IMAU80179 | GU125599 | Pickle | Sichuan | 6.62±0.39 | 36.29±0.04 | 4.71±0.11 |
| IMAU80181 | GU125601 | Pickle | Sichuan | 5.75±0.85 | 34.31±0.61 | 3.93±0.87 |
| IMAU80180 | GU125600 | Pickle | Sichuan | 3.06±0.22 | 32.76±0.93 | 2.34±0.56 |
| IMAU80170 | GU125590 | Pickle | Sichuan | 6.96±1.16 | 33.10±1.87 | 5.60±3.35 |
| IMAU80159 | GU125579 | Pickle | Sichuan | 4.76±1.40 | 33.00±1.27 | 2.65±2.79 |
| IMAU80161 | GU125581 | Pickle | Sichuan | 6.81±0.09 | 29.71±4.27 | 4.23±1.58 |
| IMAU80162 | GU125582 | Pickle | Sichuan | 8.62±0.50 | 32.06±0.47 | 2.21±0.43 |
| IMAU80160 | GU125580 | Pickle | Sichuan | 6.91±1.69 | 32.52±0.23 | 4.80±0.46 |
| IMAU80163 | GU125583 | Pickle | Sichuan | 3.54±1.39 | 30.05±2.06 | 3.41±2.35 |
| IMAU80108 | GU125530 | Pickle | Sichuan | 2.58±0.15 | 31.27±2.09 | 5.33±2.46 |
| IMAU80158 | GU125578 | Pickle | Sichuan | 2.51±0.84 | 32.52±0.84 | 7.27±1.00 |
| IMAU80188 | GU125608 | Pickle | Sichuan | 3.60±0.41 | 31.24±0.48 | 7.71±1.30 |
| IMAU80100 | GU125522 | Pickle | Sichuan | 3.11±2.04 | 30.48±1.28 | 5.63±1.80 |
| IMAU80002 | GU125424 | Pickle | Sichuan | - | 30.42±0.36 | 4.15±2.83 |
| IMAU80043 | GU125465 | Pickle | Sichuan | - | 29.78±0.84 | 3.34±0.83 |
| IMAU80007 | GU125429 | Pickle | Sichuan | 8.21±0.14 | 29.68±0.72 | 5.94±0.68 |
| IMAU80178 | GU125598 | Pickle | Sichuan | - | 26.93±3.89 | 3.12±2.18 |
| IMAU80038 | GU125460 | Pickle | Sichuan | - | 28.15±2.40 | - |
| IMAU80183 | GU125603 | Pickle | Sichuan | 7.79±0.55 | 24.77±1.20 | - |
| IMAU80013 | GU125435 | Pickle | Sichuan | - | 24.16±0.68 | - |
| IMAU80033 | GU125455 | Pickle | Sichuan | - | 22.58±1.57 | - |
| IMAU80021 | GU125443 | Pickle | Sichuan | 2.48±0.75 | 23.67±3.32 | 7.66±1.54 |
| IMAU80050 | GU125472 | Pickle | Sichuan | 10.77±1.02 | 24.50±1.57 | 4.83±5.35 |
| IMAU80031 | GU125453 | Pickle | Sichuan | - | 27.71±0.81 | 8.35±2.75 |
| IMAU80045 | GU125467 | Pickle | Sichuan | - | 24.18±2.45 | 5.24±3.26 |
| IMAU80029 | GU125451 | Pickle | Sichuan | 3.73±2.82 | 23.75±0.42 | - |
| IMAU80026 | GU125448 | Pickle | Sichuan | - | 24.15±0.97 | - |
| IMAU80051 | GU125473 | Pickle | Sichuan | 5.93±0.37 | 24.87±0.73 | 5.90±2.35 |
| IMAU80064 | GU125486 | Pickle | Sichuan | - | 29.43±1.97 | 7.18±2.31 |
| IMAU80046 | GU125468 | Pickle | Sichuan | 4.41±0.85 | 28.90±0.20 | 4.52±0.96 |
| IMAU80049 | GU125471 | Pickle | Sichuan | 6.54±0.94 | 27.43±3.52 | 5.43±1.11 |
| IMAU80028 | GU125450 | Pickle | Sichuan | 2.68±0.06 | 25.80±2.17 | 3.65±0.73 |
| IMAU80006 | GU125428 | Pickle | Sichuan | 12.82±0.35 | 23.85±0.53 | - |
| IMAU80186 | GU125606 | Pickle | Sichuan | 6.99±1.22 | 25.23±2.79 | 4.98±2.91 |
| IMAU40003 | FJ749722 | Koumiss | Qinghai | 2.17±1.62 | 20.69±3.86 | - |
| IMAU40014 | FJ749733 | Koumiss | Qinghai | - | 24.69±2.45 | - |
| IMAU40070 | FJ749345 | Fermented yak milk | Qinghai | 11.98±0.73 | 26.44±2.76 | 5.51±3.18 |
| IMAU40126 | FJ749395 | Fermented yak milk | Qinghai | - | 24.79±0.59 | 6.76±1.71 |
| IMAU40091 | FJ749366 | Fermented yak milk | Sichuan | - | 26.13±0.77 | 5.02±1.65 |
| IMAU40122 | FJ749392 | Fermented yak milk | Qinghai | - | 22.86±2.06 | - |
| IMAU10395 | HM218120 | Yoghurt | Inner Mongolia | - | 24.87±0.56 | 3.83±0.34 |
| IMAU10418 | HM218143 | Yoghurt | Inner Mongolia | - | 24.17±0.75 | 3.19±1.66 |
| IMAU60049 | FJ749774 | Fermented cow milk | Tibet | 11.28±0.71 | 25.69±2.44 | 5.18±2.88 |
| IMAU60042 | FJ211391 | Fermented cow milk | Tibet | - | 23.47±0.03 | 3.62±0.78 |
| IMAU10273 | GU138601 | Sour dough | Inner Mongolia | - | 22.59±1.77 | 3.75±2.08 |
| IMAU80070 | GU125492 | Pickle | Sichuan | 9.05±0.20 | 32.41±0.36 | 22.76±1.32 |
| IMAU70035 | GQ131151 | Sour porridge | Inner Mongolia | 8.91±0.30 | 31.83±0.62 | 22.54±2.60 |
| IMAU10324 | HM218050 | Yoghurt | Inner Mongolia | 8.91±0.21 | 31.70±0.79 | 18.14±2.43 |
| IMAU10180 | GU138508 | Sour dough | Inner Mongolia | 8.74±0.15 | 31.57±0.86 | 18.26±1.84 |
| IMAU20115 | HM057853 | Yoghurt | Mongolia | 8.74±0.21 | 31.45±0.48 | 16.90±2.35 |
| IMAU80102 | GU125524 | Pickle | Sichuan | 8.75±1.16 | 31.27±1.29 | 14.58±7.43 |
| IMAU80076 | GU125498 | Pickle | Sichuan | 9.20±0.39 | 31.01±1.32 | 21.12±2.77 |
| IMAU10267 | GU138595 | Sour dough | Inner Mongolia | 7.89±0.76 | 30.93±0.99 | 18.80±2.26 |
| IMAU10191 | GU138519 | Sour dough | Inner Mongolia | 8.37±0.16 | 30.87±1.47 | 16.72±1.24 |
| IMAU10265 | GU138593 | Sour dough | Inner Mongolia | 8.18±0.50 | 30.74±1.57 | 16.40±0.54 |
| IMAU10608 | HM218332 | Koumiss | Inner Mongolia | 8.39±0.48 | 30.19±1.45 | 17.79±1.01 |
| IMAU10263 | GU138591 | Sour dough | Inner Mongolia | 10.29±0.92 | 24.93±0.30 | 20.84±0.71 |
| IMAU80597 | HM058789 | Qula | Gansu | 9.22±0.71 | 22.55±1.77 | 15.26±0.73 |
| IMAU80297 | HM058577 | Yak milk | Sichuan | 10.67±0.18 | 23.47±1.46 | 15.35±2.65 |
| IMAU80325 | HM058605 | Yak milk | Sichuan | 6.57±0.56 | 18.87±0.85 | 8.14±2.51 |
| IMAU80441 | HM058694 | Yak milk | Sichuan | 7.23±1.11 | 16.19±1.84 | 6.89±0.03 |
| IMAU10586 | HM218310 | Koumiss | Inner Mongolia | 7.66±0.87 | 15.52±7.67 | 9.61±0.41 |
| IMAU10374 | HM218100 | Yoghurt | Inner Mongolia | 9.03±0.14 | 16.47±1.45 | 3.71±0.59 |
| IMAU10386 | HM218111 | Yoghurt | Inner Mongolia | 9.03±0.49 | 25.05±1.70 | 10.94±0.66 |
| IMAU10942 | HM218649 | Yoghurt | Inner Mongolia | 9.64±0.34 | 20.42±5.01 | 5.09±1.19 |
| IMAU11029 | HM218730 | Yoghurt | Inner Mongolia | 8.65±0.28 | 18.65±1.08 | 7.71±1.54 |
| IMAU10567 | HM218292 | Yoghurt | Inner Mongolia | 9.11±0.19 | 19.49±2.46 | 8.77±0.78 |
| IMAU10969 | HM218673 | Yoghurt | Inner Mongolia | 9.42±0.17 | 17.96±5.47 | 11.39±1.59 |
| IMAU10578 | HM218303 | Koumiss | Inner Mongolia | 8.97±0.47 | 18.71±2.85 | 9.25±2.79 |
| IMAU10570 | HM218295 | Koumiss | Inner Mongolia | 8.03±0.31 | 16.75±1.76 | 7.31±1.64 |
| IMAU10574 | HM218299 | Koumiss | Inner Mongolia | 10.79±4.59 | 14.22±7.88 | - |
| IMAU10566 | HM218291 | Koumiss | Inner Mongolia | 12.12±1.31 | 15.85±1.88 | 18.00±0.37 |
| IMAU10576 | HM218301 | Koumiss | Inner Mongolia | 4.97±2.13 | 15.14±1.59 | 4.08±0.73 |
| IMAU11038 | HM218739 | Koumiss | Inner Mongolia | 7.96±0.95 | 28.20±3.48 | 10.59±4.19 |
| IMAU10572 | HM218297 | Koumiss | Inner Mongolia | 6.60±1.15 | 26.79±3.44 | 4.61±3.45 |
| IMAU10591 | HM218315 | Koumiss | Inner Mongolia | 7.59±0.26 | 28.78±1.01 | 7.25±0.64 |
| IMAU10596 | HM218320 | Koumiss | Inner Mongolia | 7.32±0.64 | 27.25±2.25 | 7.82±0.94 |
| IMAU80088 | GU125510 | Pickle | Sichuan | 6.94±0.23 | 25.85±0.80 | 2.87±0.00 |
| IMAU80092 | GU125514 | Pickle | Sichuan | 6.54±1.01 | 23.87±4.43 | 6.64±0.79 |
| IMAU80090 | GU125512 | Pickle | Sichuan | 4.34±0.20 | 18.73±0.28 | - |
| IMAU80097 | GU125519 | Pickle | Sichuan | 7.63±0.39 | 28.13±1.57 | 6.36±2.23 |
| IMAU80078 | GU125500 | Pickle | Sichuan | 7.86±0.37 | 27.03±0.71 | 8.24±1.51 |
| IMAU80072 | GU125494 | Pickle | Sichuan | 7.49±0.18 | 24.28±2.27 | 8.65±2.06 |
| IMAU80095 | GU125517 | Pickle | Sichuan | 6.73±0.43 | 17.59±5.71 | 2.85±0.00 |
| IMAU80101 | GU125523 | Pickle | Sichuan | 8.28±0.26 | 27.66±1.39 | 11.79±3.58 |
| IMAU80104 | GU125526 | Pickle | Sichuan | 7.78±0.39 | 18.96±9.04 | 8.60±3.50 |
| IMAU10258 | GU138586 | Sour dough | Inner Mongolia | 8.29±0.07 | 29.28±1.16 | 8.73±0.38 |
| IMAU10247 | GU138575 | Sour dough | Inner Mongolia | 7.13±0.05 | 28.45±1.41 | 4.11±0.06 |
| IMAU20020 | FJ844949 | Koumiss | Mongolia | 7.64±0.67 | 27.75±3.14 | 10.35±3.79 |
| IMAU10285 | GU138613 | Sour dough | Inner Mongolia | 10.00±0.98 | 27.74±5.44 | 23.73±2.63 |
| IMAU10282 | GU138610 | Sour dough | Inner Mongolia | 7.59±0.53 | 28.63±2.44 | 13.12±3.90 |
| IMAU20113 | HM057851 | Yoghurt | Mongolia | 10.4±0.09 | 26.22±0.88 | 14.89±2.32 |
| IMAU80016 | GU125438 | Pickle | Sichuan | 8.93±0.69 | 21.88±1.29 | 6.77±0.39 |
| IMAU21301 | GU125481 | Pickle | Sichuan | 9.04±5.01 | 18.52±0.90 | 11.86±2.10 |
| IMAU80093 | GU125515 | Pickle | Sichuan | 8.94±0.23 | 24.41±0.49 | 7.84±1.87 |
| IMAU80094 | GU125516 | Pickle | Sichuan | 7.93±0.50 | 20.04±0.93 | - |
| IMAU80151 | GU125573 | Pickle | Sichuan | 8.39±0.65 | 21.21±2.97 | 10.79±1.41 |
| IMAU80153 | GU125575 | Pickle | Sichuan | 9.75±0.28 | 24.42±0.63 | 12.82±0.68 |
| IMAU80107 | GU125529 | Pickle | Sichuan | 9.68±0.37 | 24.02±2.39 | 13.43±0.95 |
| IMAU80099 | GU125521 | Pickle | Sichuan | 8.77±0.43 | 21.11±2.92 | 11.14±0.00 |
| IMAU80115 | GU125537 | Pickle | Sichuan | 10.07±0.39 | 22.99±2.26 | 15.48±4.09 |

^1^ Data are expressed in mean ± SD.

^2^ ‘-’ represents no detectable degradation activity.
