# Supplementary material for: Screening for Lactobacillus plantarum Strains That Possess Organophosphorus Pesticide-Degrading Activity and Metabolomic Analysis of Phorate Degradation
Source: Front Microbiol. 2018 Sep 3;9:2048. doi: 10.3389/fmicb.2018.02048 (PMC6130228; doi:10.3389/fmicb.2018.02048)
Supplement: Supplementary file 2 [file Table_2.docx]

**Supplement Table 2** Pearson correlation coefficients between the OPPs degradation and acid phosphatase activity.

| Degradation rate | Phosphatase activity (%) | |
| --- | --- | --- |
|  | Intracellular | Extracellular |
| Omethoate | 0.48 | 0.13 |
| Phorate | -0.63 | 0.12 |
| Dimethoate | -0.44 | -0.06 |

^1^ No significant correlation (P > 0.05) existed between the degradation rate of all three investigated OPPs and acid phosphatase activity (both intracellular and extracellular).
