# Supplementary material for: Screening for Lactobacillus plantarum Strains That Possess Organophosphorus Pesticide-Degrading Activity and Metabolomic Analysis of Phorate Degradation
Source: Front Microbiol. 2018 Sep 3;9:2048. doi: 10.3389/fmicb.2018.02048 (PMC6130228; doi:10.3389/fmicb.2018.02048)
Supplement: Supplementary file 3 [file Image_1.pdf]

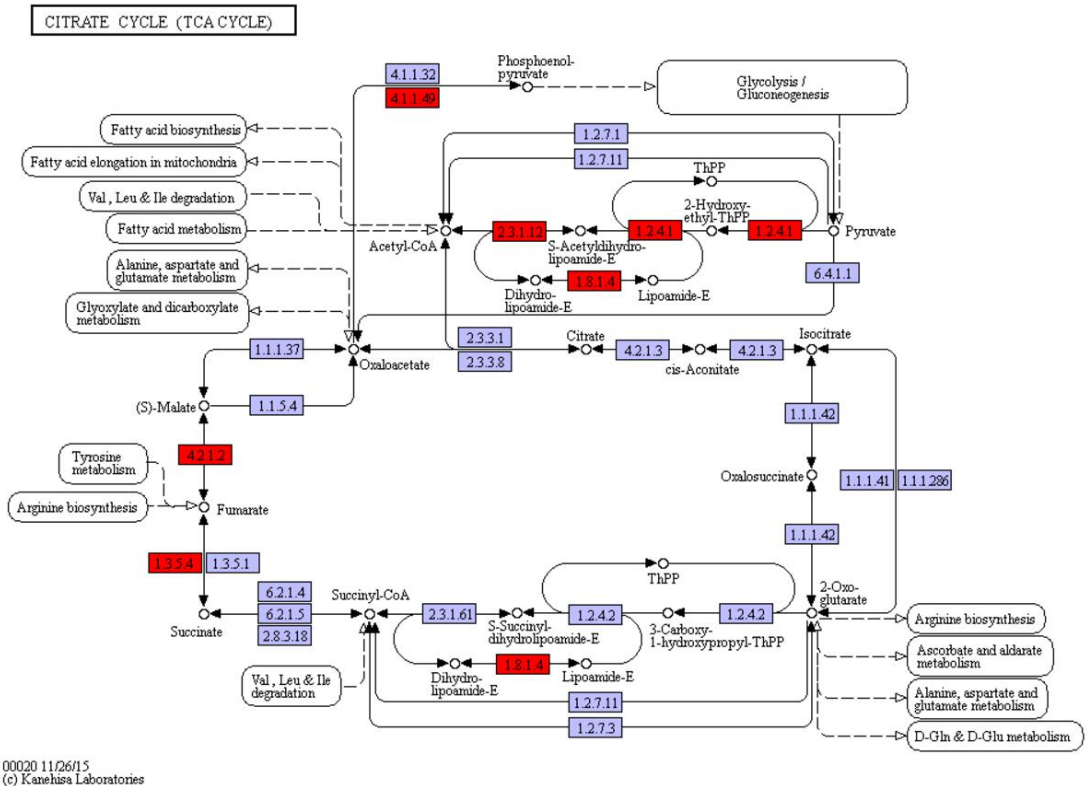

Supplement Figure 1. The partial citrate cycle of *Lactobacillus plantarum* P9 predicted based on whole genome analysis. Putative enzymes that are coded in the *Lactobacillus plantarum* IMAU70010 genome are shaded red, including fumarate reductase flavoprotein subunit (EC:1.3.5.4), dihydrolipoamide dehydrogenase (EC:1.8.1.4), fumarate hydratase, class I (EC:4.2.1.2), pyruvate dehydrogenase E1 component (EC:1.2.4.1), pyruvate dehydrogenase E2 component (EC:2.3.1.12), and phosphoenolpyruvate carboxykinase (ATP) (EC: 4.1.1.49).
