# Supplementary material for: Screening for Lactobacillus plantarum Strains That Possess Organophosphorus Pesticide-Degrading Activity and Metabolomic Analysis of Phorate Degradation
Source: Front Microbiol. 2018 Sep 3;9:2048. doi: 10.3389/fmicb.2018.02048 (PMC6130228; doi:10.3389/fmicb.2018.02048)
Supplement: Supplementary file 4 [file Image_2.pdf]

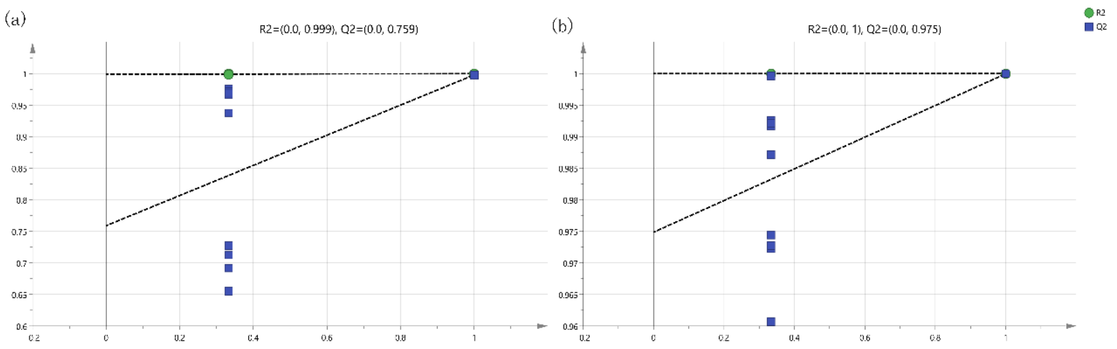

Supplement Figure 2 Permutation test performed with 200 random permutations in an OPLS-DA model showing R^2 (green dot) and Q^2 (blue square) values from the permuted analysis (bottom left), which were lower than that of the corresponding original values (top right). “a” and “b” represent the permutation of metabolomic data generated in positive and negative ion modes, respectively.
